# Diagnostic accuracy of endocytoscopy via artificial intelligence in colorectal lesions: A systematic review and meta‑analysis

**DOI:** 10.1371/journal.pone.0294930

**Published:** 2023-12-19

**Authors:** Hangbin Zhang, Xinyu Yang, Ye Tao, Xinyi Zhang, Xuan Huang

**Affiliations:** Department of Gastroenterology, The First Affiliated Hospital of Zhejiang Chinese Medical University, Hangzhou, China; Dalin Tzu Chi Hospital, Buddhist Tzu Chi Medical Foundation, TAIWAN

## Abstract

**Background:**

Endocytoscopy (EC) is a nuclei and micro-vessels visualization in real-time and can facilitate "optical biopsy" and "virtual histology" of colorectal lesions. This study aimed to investigate the significance of employing artificial intelligence (AI) in the field of endoscopy, specifically in diagnosing colorectal lesions. The research was conducted under the supervision of experienced professionals and trainees.

**Methods:**

EMBASE, PubMed, Cochrane Library, Web of Science, Chinese National Knowledge Infrastructure (CNKI) database, and other potential databases were surveyed for articles related to the EC with AI published before September 2023. RevMan (5.40), Stata (14.0), and R software (4.1.0) were used for statistical assessment. Studies that measured the accuracy of EC using AI for colorectal lesions were included. Two authors independently assessed the selected studies and their extracted data. This included information such as the country, literature, total study population, study design, characteristics of the fundamental study and control groups, sensitivity, number of samples, assay methodology, specificity, true positives or negatives, and false positives or negatives. The diagnostic accuracy of EC by AI was determined by a bivariate random-effects model, avoiding a high heterogeneity effect. The ANOVA model was employed to determine the more effective approach.

**Results:**

A total of 223 studies were reviewed; 8 articles were selected that included 2984 patients (4241 lesions) for systematic review and meta-analysis. AI assessed 4069 lesions; experts diagnosed 3165 and 5014 by trainees. AI demonstrated high accuracy, sensitivity, and specificity levels in detecting colorectal lesions, with values of 0.93 (95% CI: 0.90, 0.95) and 0.94 (95% CI: 0.73, 0.99). Expert diagnosis was 0.90 (95% CI: 0.85, 0.94), 0.87 (95% CI: 0.78, 0.93), and trainee diagnosis was 0.74 (95% CI: 0.67, 0.79), 0.72 (95% CI: 0.62, 0.80). With the EC by AI, the AUC from SROC was 0.95 (95% CI: 0.93, 0.97), therefore classified as excellent category, expert showed 0.95 (95% CI: 0.93, 0.97), and the trainee had 0.79 (95% CI: 0.75, 0.82). The superior index from the ANOVA model was 4.00 (1.15,5.00), 2.00 (1.15,5.00), and 0.20 (0.20,0.20), respectively. The examiners conducted meta-regression and subgroup analyses to evaluate the presence of heterogeneity. The findings of these investigations suggest that the utilization of NBI technology was correlated with variability in sensitivity and specificity. There was a lack of solid evidence indicating the presence of publishing bias.

**Conclusions:**

The present findings indicate that using AI in EC can potentially enhance the efficiency of diagnosing colorectal abnormalities. As a valuable instrument, it can enhance prognostic outcomes in ordinary EC procedures, exhibiting superior diagnostic accuracy compared to trainee-level endoscopists and demonstrating comparability to expert endoscopists. The research is subject to certain constraints, namely a limited number of clinical investigations and variations in the methodologies used for identification. Consequently, it is imperative to conduct comprehensive and extensive research to enhance the precision of diagnostic procedures.

## Introduction

Colorectal lesions, often known as polyps, are the most common occurrences in this area of medicine. Based on their histological characteristics, these polyps are classified into two types: neoplastic and non-neoplastic. The guidelines provided by the American Society for Gastrointestinal Endoscopy (ASGE) and the European Society for Gastrointestinal Endoscopy (ESGE) urge the excision of all neoplastic colorectal polyps. It is important to highlight that the World Health Organization (WHO) classification of hyperplastic polyps falls within the broader category of sessile serrated lesions. Polyps that exhibit hyperplasia and have a size smaller than 5mm are the sole anomaly within the category of polyps commonly considered to have a predisposition for malignancy. To effectively reduce the incidence of colorectal cancer (CRC) and enhance long-term survival rates, it is crucial to prioritize the implementation of endoscopic clearance procedures and histological testing for all premalignant polyps [1, 2]. However, because most of the colon polyps are hyperplastic (10%-35% in Western populations), they are left un-resected due to cost and risk of adverse situations; wrong removal can cause multiple complications, such as perforation (1.7/1000) and bleeding (22.3/100) [3]; therefore, real-time neoplastic differentiation in these polyps is required for resection. ESGE and ASGE suggest optical diagnosis is a promising strategy for diminutive colorectal polyps as it is cost-effective and reduces the risks associated with polypectomy [4]. CRC has a high mortality rate globally [5], so prevention is essential. The lesions are usually missed because of the poor skills of the endoscopist and bowel movement status [6]; the lesions’ shape and anatomy also affect their diagnosis. Blind spots and lesions that are flat or depressed might be frequently overlooked.

Endocytoscopy (EC; Olympus Co. Ltd) is a novel technique carried out by an endoscopic system comprising a contact light microscope attached to a conventional colonoscope’s distal tip [7, 8]. The device enables magnification of 520 times, and when used in conjunction with methylene blue staining, EC can produce images that closely resemble those obtained by histological examination. As a result, the application of this technique has the potential to improve the accuracy of optical diagnostics significantly. Based on the results obtained from a randomized controlled study, it was shown that the application of an optical biopsy method designated EC exhibited a similar degree of precision (94.1%) when compared to that of a traditional biopsy (96.5%) in effectively discerning malignant polyps [9]. Recent Artificial Intelligence (AI) breakthroughs have significantly advanced endocytoscopic imaging and results interpretation by suggesting polyp histopathology during EC. Ultra-magnified microvessels within the lesion are detectable during EC; this technique has been utilized to show intestinal mucosal tissue and live cells *in vivo* in real-time, and it consistently detects the histopathology of gastrointestinal tract lesions [10, 11]. AI is described as computers’ ability to carry out tasks that usually need human intelligence, and therefore, it mimics the cognitive activity of humans. Recently, real-time computer-aided diagnosis (CAD) has become very popular for endoscopic imaging as it has more accuracy and reduces inter-observer variability in optical colorectal lesion diagnosis [12, 13]. The current protocol involves using neural networks, most commonly deep and convolutional neural networks. These networks can autonomously isolate and learn characters from the "big data" of healthcare [14]. In the field of EC, AI is anticipated to have two crucial functions associated with colonoscopy practice: polyp detection and its characterization [15, 16]. For surveillance intervals after the polypectomy and recto-sigmoid polyps showing adenomatous histology, Preservation and Incorporation of Valuable Endoscopic Innovations (PIVI) recommend a ≥ 90% agreement rate and ≥ 90% negative predictive value. However, it is challenging to meet this criterion for real-time endoscopic histologic evaluation of diminutive polyps [17]. Owing to its cost-efficiency, the accessibility, regulation, and effective EC via AI implementation require attention.

Introducing a reliable method to differentiate neoplastic polyps from non-neoplastic polyps is crucial to minimize resource wastage, over-diagnosis, and the potential for consequences. This necessitates prompt action. The existing body of research on the diagnostic precision of AI in identifying colorectal lesions by EC indicates a lack of conclusive evidence. To mitigate this discrepancy, the current study conducted an extensive review and meta-analysis of case-control studies to investigate the correlation between EC utilization and the occurrence of AI and colorectal lesions.

## Methods

This investigation followed the parameters of Preferred Reporting Items for Systematic Reviews and Meta-Analyses (PRISMA) [18] and was also submitted in PROSPERO (CRD42023388421).

### Search strategy

The published articles from inception to September 2023 were searched on EMBASE, Cochrane Library, Web of Science, PubMed, and Chinese databases of Chinese National Knowledge Infrastructure (CNKI) using keywords’ endocytoscopy,’ ’endocytoscopic,’ ’colorectal lesions,’ ’colon lesions,’ ’Artificial Intelligence,’ and ’computer-aided diagnosis,’. The supplementary material contains comprehensive information regarding the outcomes of the literature search. In addition to the literature provided, references were examined to support any articles that may have been unintentionally missed. Two examiners performed literature screening independently, following a sequential process that involved initial screening, full-text evaluation, and further procedures. The corresponding authors, responsible for the final decision, resolved any discrepancies in article selection.

### Inclusion criteria

Articles that had: (1) a case-control or cohort design; (2) aimed to determine the value of EC with AI for diagnosing and/or distinguishing colorectal lesions; (3) a 2 × 2 contingency table of true negatives (TN) and false negatives (FN) or false positives (FP) and true positives (TP); (4) provided the number of data or could be calculated from the published data, were selected; (5) A comprehensive histopathological examination was conducted on all observed lesions, and the subsequent findings were employed as the established reference.

### Exclusion criteria

Articles which was not published, those which were ecological research, or lacked abstracts, reviews, letters, and comments were not included. Articles with reports, poor quality, study design defects, incomplete data, and no AI group were excluded.

### Data extraction

Two investigators independently extracted (1) the surname of the first author, (2) the average age of the participant, (3) the sample size, (4) the study design, (5) the origin country, (6) the year of publication; (7) sex of the samples; (8) specificity, sensitivity, TP, FN, FP, and TN. In case of disagreement, the corresponding authors were approached.

### Risk of bias assessment

With the help of the Quality Assessment of Diagnostic Accuracy Studies (QUADAS-2 tool), the risk of research bias and the suitability of diagnostic criteria were assessed [19]. QUADAS-2 involved four variables: standard reference, index assay, patient selection, and timing and flow. These variables elucidated the risk of selected literature bias. The initial three criteria were additionally employed in the determination of clinical implementation. For more detailed assessment criteria, please consult the provided references. Two independent investigators evaluated quality independently, and the authors responsible for the study were contacted in case of disagreements.

### Statistical methods

The quality of the data in the literature included in this study was evaluated using RevMan 5.40 software. Statistical measurements were conducted using the MIDAS module of STATA14.0. A P < 0.05 was deemed statistically significant. Specificity, sensitivity, and summary receiver operator characteristics (SROC) were evaluated and compiled via the bivariate random-effects model [20], avoiding a high heterogeneity effect. The SROC curve calculated the overall CAD diagnostic performance for colorectal lesions. A preliminary method for classifying diagnostic tests’ accuracy was based on Area Under Curve (AUC). The AUC classification criteria were: 0.90–1 = excellent, 0.80–0.90 = good, 0.70–0.80 = fair, 0.60–0.70 = poor, and 0.50–0.60 = failure [21]. Diagnostic test accuracy Mesh meta-analysis ANOVA model [22] was assessed by R software 4.1.0 (rstan 2.21.7, rtools 40) to determine a better method. The Q-test and I2 index were employed to elucidate heterogeneity in inter-study. The Q-test (p< 0.05) and *I*^*2*^ index ≥50% both revealed the presence of moderate heterogeneity, suggesting the need for further examination and discussion regarding its underlying factors. Furthermore, there is a potential presence of threshold effects concerning the proportion of heterogeneity. The investigation of potential heterogeneity was conducted by subgroup and univariate meta-regression analyses, considering several parameters such as the study’s process, type, sample size, magnification, and the utilization of narrow-band imaging (NBI) technology. If the symmetry assessment conducted by Deek demonstrated a p-value less than 0.05, it would be deemed that the publication exhibited bias.

## Results

### Study selection

**Fig 1:**
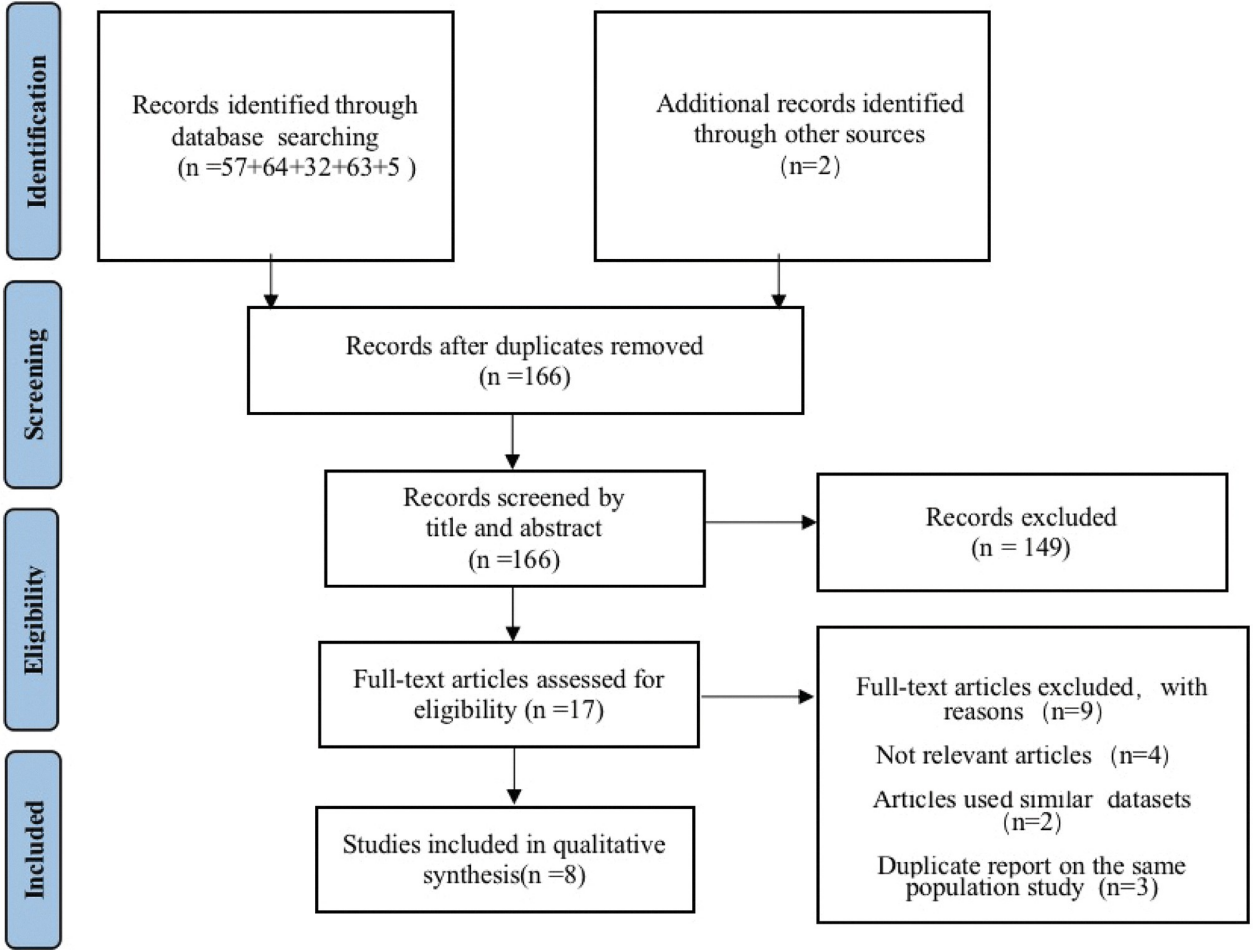
Schematics representation of the study procedures.

The flowchart depicted in Fig 1 outlines the methods utilized for conducting the literature evaluation within the scope of the present study. A complete collection of 223 articles was acquired through querying online databases and conducting manual searches. After an initial screening procedure, a total of 57 articles found to be duplicates were eliminated from the analysis. Following that, a thorough examination was conducted of the titles and abstracts of each paper, resulting in the exclusion of an additional 149 investigations. A total of four papers were considered irrelevant, whereas two studies were excluded due to the absence of comparable datasets.

Additionally, two studies were excluded since they did not provide the necessary information regarding the confidence interval for risk calculation. Lastly, 8 articles [23–30] reporting detailed figures of EC via AI in colorectal lesions were selected (Table 1). These were published between 2014 and 2022, were all in English language, and were all conducted in Japan. The study encompassed a total of 2984 individuals, with a collective number of 4241 lesions. Among these lesions, 4069 were tested using AI, qualified professionals assessed 3165, and trainees evaluated 5014 cases. The lesions were accompanied by specific data encompassing their dimensions, morphology, site, and histopathological characteristics. Table 2 presents the diagnostic accuracy statistics of EC by AI in colorectal lesions. Four articles employed the EndoBRAIN® technology, while an additional four publications utilized the CAD system for EC (EC-CAD). Only three research included data from the AI group [28–30].

**Table 1:** Characteristics of the selected literature.

Author	Year	Country	Study type	No. of lesions	Age	Sex male%	Size (mm)	Location (n)	Shape	Histopathology
Masashi Misawa	2017	Japan	Retrospective	124	65.2 ± 10.6	36/58	8.7 ± 8.8	Right 27; Left 25; Rectum 11	Protruded 20; Flat 37; Depressed 4; Type 2 2	Non-neoplastic Hyperplastic polyp 15; Low-grade adenoma 39 NeoplasticHigh-grade adenoma 3; Invasive cancer 7
Shin-ei Kudo	2019	Japan	Prospective	2000	66.3 (9.8)	63/89	4 (3–5)	Right colon 38 (38.0); Left colon 30 (30.0); Rectum 32 (32.0)	Polypoid (Is, Ip) 40 (40.0); Slightly elevated (IIa) 60 (60.0)	Non-neoplastic, n (%) Hyperplastic polyp 34 (34.0); Inflammatory polyp 1 (1.0) Neoplastic, n (%)Tubular adenoma 63 (63.0); Tubulo-villous adenoma 2 (2.0)
Yuichi Mori	2014	Japan	Retrospective	176	64.2±12.1	107/152	6.3 (2.4)	Right colon 99 (48);Left colon 73 (36);Rectum 33 (16)	Polypoid (Is, Ip) 97 (55.2); Slightly elevated (IIa) 71 (40.3); Slightly depressed (IIc, IIaþIIc) 8 (4.5)	Non-neoplastic, Hyperplastic polyp 30 (17.0); Inflammatory polyp 5 (2.8); Juvenile polyp 4 (2.3) Neoplastic, Low-grade adenoma 104 (59.1); High-grade adenoma 26 (14.8); Invasive cancer 7 (4.0)
Yuichi Mori	2016	Japan	Prospective	139	65(10)	84/134	5(2)	Right colon 77 (43.7);Left colon 66 (37.5);Rectum 33 (18.8)	Polypoid (Is, Ip) 86 (42); Slightly elevated (IIa) 112(55); Slightly depressed (IIc, IIaþIIc) 7 (3)	Non-neoplastic, Hyperplastic polyp 30 (17.0); Inflammatory polyp 5 (2.8); Juvenile polyp 4 (2.3) Neoplastic, Low-grade adenoma 104 (59.1); High-grade adenoma 26 (14.8); Invasive cancer 7 (4.0)
Yuichi Mori	2018	Japan	Prospective	450	67 (58–73)	235/325	3 (3–4)	Cecum 28 (6.0); Ascending colon 81 (17.4); Transverse colon 78 (17.0); Descending colon 29 (6.2); Sigmoid colon 137 (29.4); Rectum 113 (24.2)	Polypoid (Is and Ip) 105 (22.5); Slightly elevated (IIa) 360 (77.3); Slightly depressed (IIc) 1 (0.2)	Neoplastic 282 (60.5); Nonneoplastic 176 (37.8); Nonanalyzable† 8 (1.7)
Kenichi Takeda	2017	Japan	Retrospective	375	Adenoma 65.3 ± 11.7 Invasive cancer 64.2 ± 11.2	45/76	Adenoma 11.0 ± 9.5 Invasive cancer 30.8 ± 14.3	Cecum 7; Ascending colon 9; Transverse colon 19; Descending colon 6; Sigmoid colon 24; Rectum 11	Polypoid (Is, Isp, Ip) 36; Slightly elevated (IIa); 35 Slightly depressed (IIc, IIa +IIc, Is +IIc)5	Low grade adenoma 48; High grade adenoma 6; Invasive cancer 22
Masashi Misawa	2016	Japan	Retrospective	85	63.8±12.0	20/33	8.6 ± 10.3	Right colon 15; Left colon 10; Rectum 11	Protruded 9; Flat elevated 26; Depressed 1	Nonneoplasms, Hyperplastic polyp 17 Neoplasms, Low-grade adenoma 14; High-grade adenoma 3; Invasive carcinoma 2
Ishita Barua	2022	Japan	Prospective	892	67 (60–74)	327/518	Neoplastic Polyps 4(3–5) Nonneoplastic Polyps 3 (2–3)	Neoplastic, Sigmoid colon 274 (76.3); Rectum 85 (23.7) Nonneoplastic, Sigmoid colon 260 (48.8);Rectum 273 (51.2)	Neoplastic, Polypoid (Is, Ip) 175 (48.7); Nonpolypoid (type IIa) 184 (51.3) Non-neoplastic, Polypoid (Is, Ip) 109 (20.5); Nonpolypoid (type IIa) 424 (79.5)	Non-neoplastic, Hyperplastic polyp 485 (91.0); Inflammatory polyp 8 (1.5); Other 40 (7.5) Neoplastic, Low-grade adenoma 335 (93.3); High-grade adenoma 5 (1.4); sessile serrated 19 (5.3)

**Table 2:** The diagnostic accuracy data of endocytoscopy using artificial intelligence in colorectal lesions.

Author	Magnification	Scope	Method	CAD				Experts				Trainees			
				TP	FP	FN	TN	TP	FP	FN	TN	TP	FP	FN	TN
Masashi Misawa	380X	NBI	EndoBRAIN®	84	10	5	25	318	25	53	100	158	37	99	78
Shin-ei Kudo	520X	NBI	EndoBRAIN®	1260	0	40	700	603	20	20	330	920	240	380	460
Yuichi Mori	380X	WLI	EC-CAD 1st	126	8	11	31	242	26	32	52	228	34	46	44
Yuichi Mori	380X	WLI	EC-CAD 2nd	80	4	11	44	248	16	25	128	646	106	264	374
Yuichi Mori	520X	NBI	EC-CAD 2nd	262	15	17	156	476	26	82	316	439	50	119	292
Kenichi Takeda	380X	NBI	EC-CAD 1st	68	1	8	90								
Masashi Misawa	380X	NBI	EndoBRAIN®	49	1	9	41								
Ishita Barua	520X	NBI	EndoBRAIN®	335	74	35	448								

### Quality assessment

**Fig 2:**
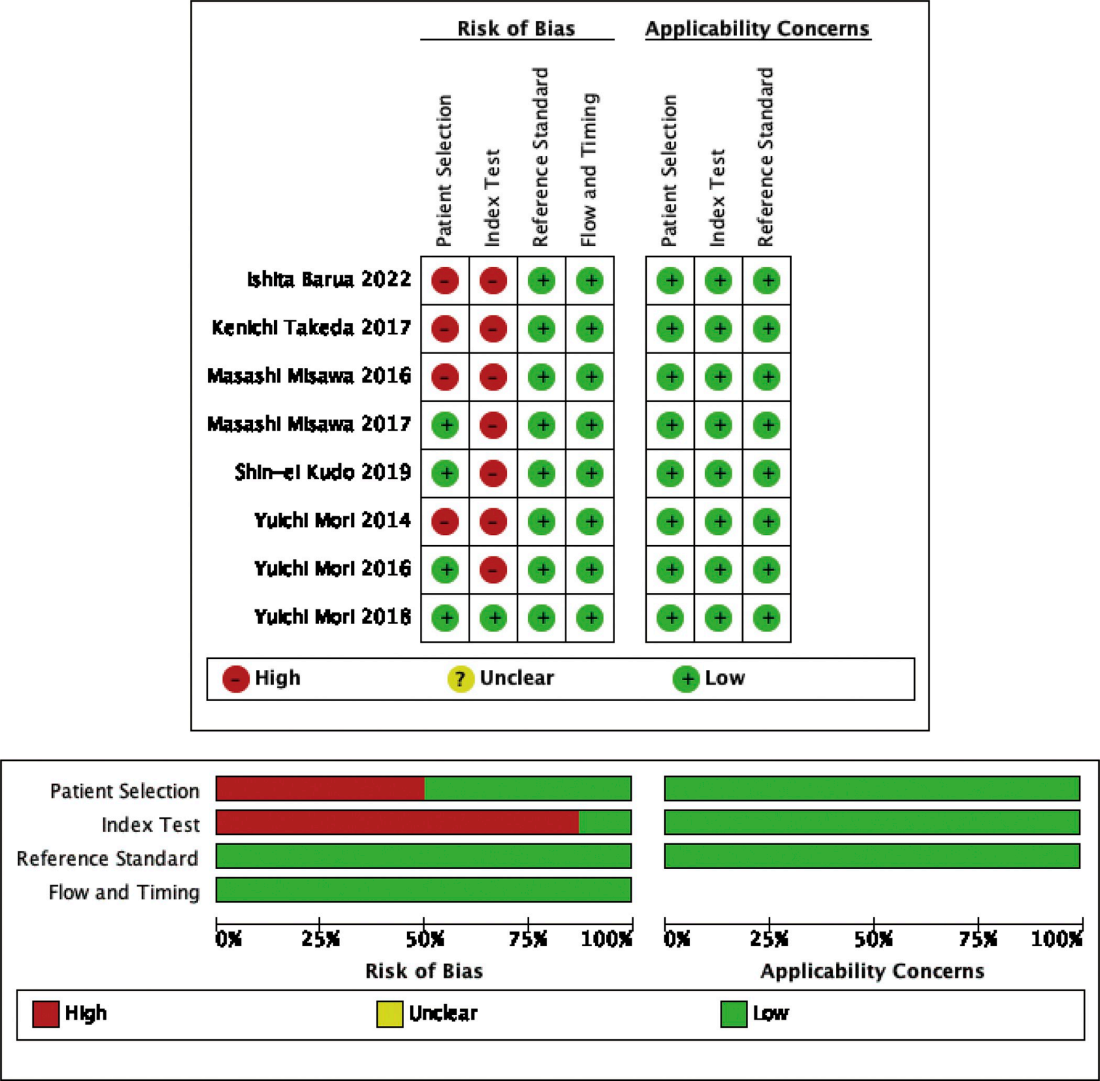
Risk of bias evaluation.

Fig 2 demonstrates the highlighted bias risk outcomes and the suitability of the selected articles. The research revealed a low-risk score in terms of applicability problems. Yuichi Mori 2018 had the lowest bias risk and applicability concern in all domains; the remaining 7 articles had high "index test" category scores. 4 studies scored high in the "patient selection" category. Overall, the biased risk of the selected research had high applicability, acceptable range, and followed QUADAS-2 assessment criteria.

### Diagnostic accuracy

**Fig 3:**
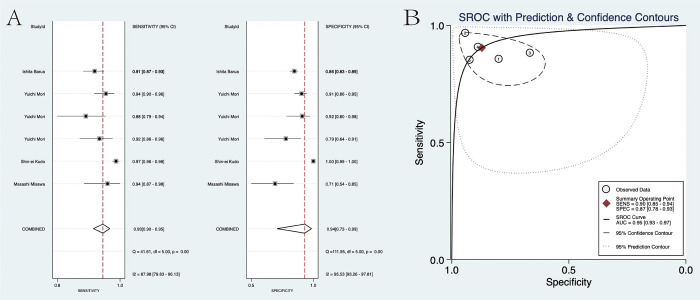
(A) The pooled specificity and sensitivity for the AI group. (B) The SROC curve for the AI group.

**Fig 4:**
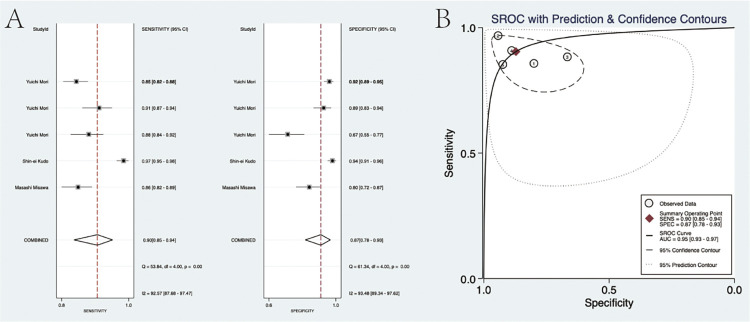
(A) The pooled specificity and sensitivity of the Expert cohort. (B) The SROC curve for the Expert cohort.

**Fig 5:**
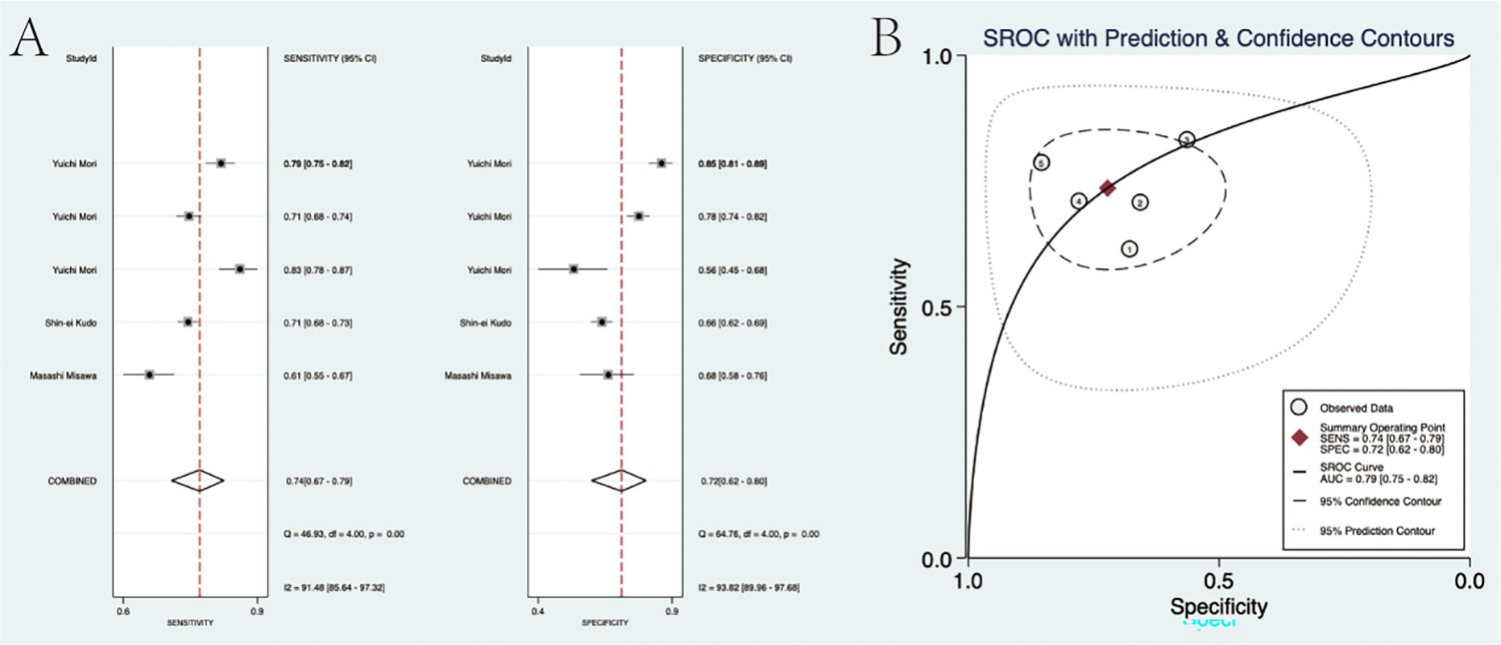
(A) The pooled specificity and sensitivity of the Trainee cohort. (B) The SROC curve for the Trainee cohort.

**Fig 6:**
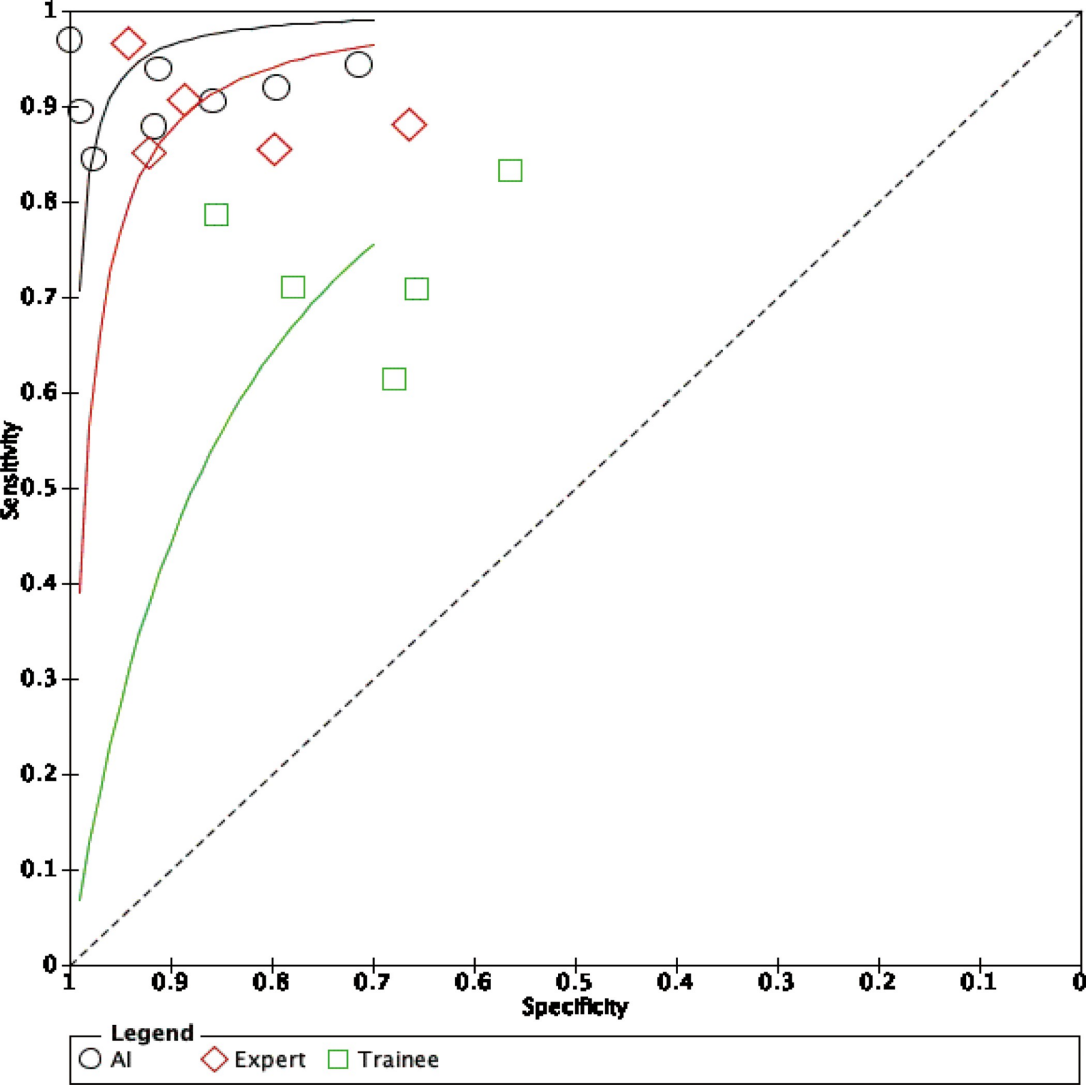
The SROC curve for AI, expert, and trainee.

For the diagnostic value of EC via AI for colorectal lesions, the specificity and sensitivity were 0.94 (95% CI: 0.73, 0.99) and 0.93 (95%CI: 0.90, 0.95), the area under the SROC curve (AUSROC) of CAD was 0.95 (95% CI: 0.93, 0.97) (Fig 3). The specificity and sensitivity of experts were 0.87 (95% CI: 0.78, 0.93) and 0.90 (95%CI: 0.85, 0.94), the AUSROC curve was 0.95 (95% CI: 0.93, 0.97) (Fig 4). The specificity and sensitivity of trainees were 0.72 (95% CI: 0.62, 0.80) and 0.74 (95%CI: 0.67, 0.79), the AUSROC curve was 0.79 (95% CI: 0.75, 0.82) (Fig 5). SROC curves for AI, expert, and trainee were also established (Fig 6). The ANOVA model test demonstrated a higher diagnostic accuracy with index values of 4.00 (95% CI: 1.15–5.00), 2.00 (95% CI: 1.15–5.00), and 0.20 (95% CI: 0.20–0.20), as presented in Table 3. The supplementary material contains complete details regarding the outcomes of the ANOVA model analysis. There was significant heterogeneity among the three methods.

**Table 3:** Accuracy of diagnostic test results of mesh meta-analysis ANOVA model.

Method	Sensitivity	Specificity	DOR	S	RSEN	RSPE
AI	0.95 (0.93,0.97)	0.85 (0.81,0.89)	113.90 (31.34,184.36)	4.76 (3.00,5.00)	1.12 (1.10,1.15)	1.54 (1.44,1.66)
Expert	0.94 (0.93,0.95)	0.77 (0.74,0.80)	50.55 (6.19,63.99)	1.25 (1.00,3.00)	1.11 (1.08,1.13)	1.39 (1.31,1.48)
Trainee	0.85 (0.83,0.86)	0.56 (0.53,0.59)	6.84 (5.87,8.16)	0.20 (0.20,0.20)	1	1

S, superior index; RSEN, Relative sensitivity; RSPE, Relative specificity.

**Fig 7:**
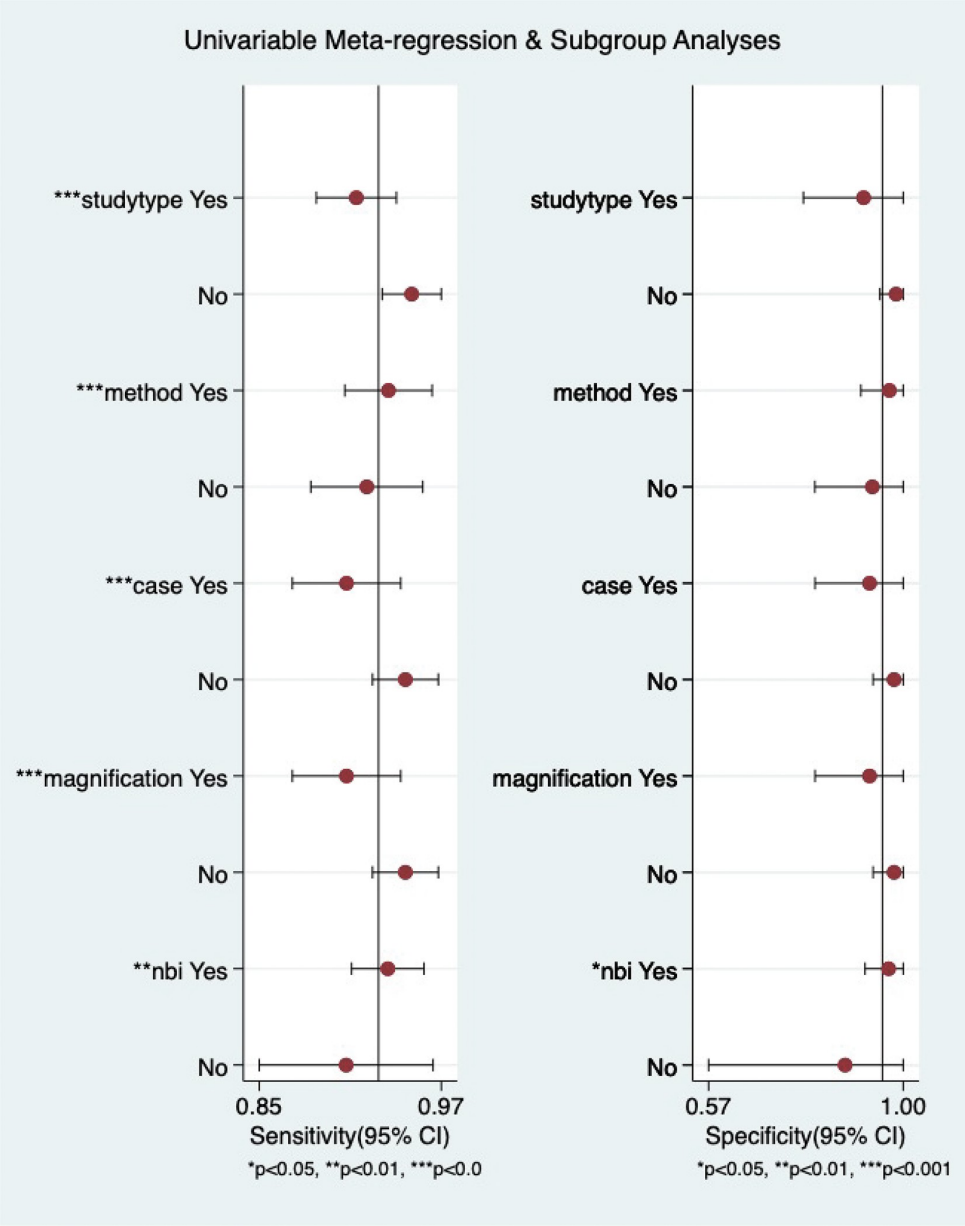
Univariate meta-regression and subgroup analyses in colorectal lesion diagnosis. *P < 0.05, **P < 0.01, ***P < 0.001.

**Fig 8:**
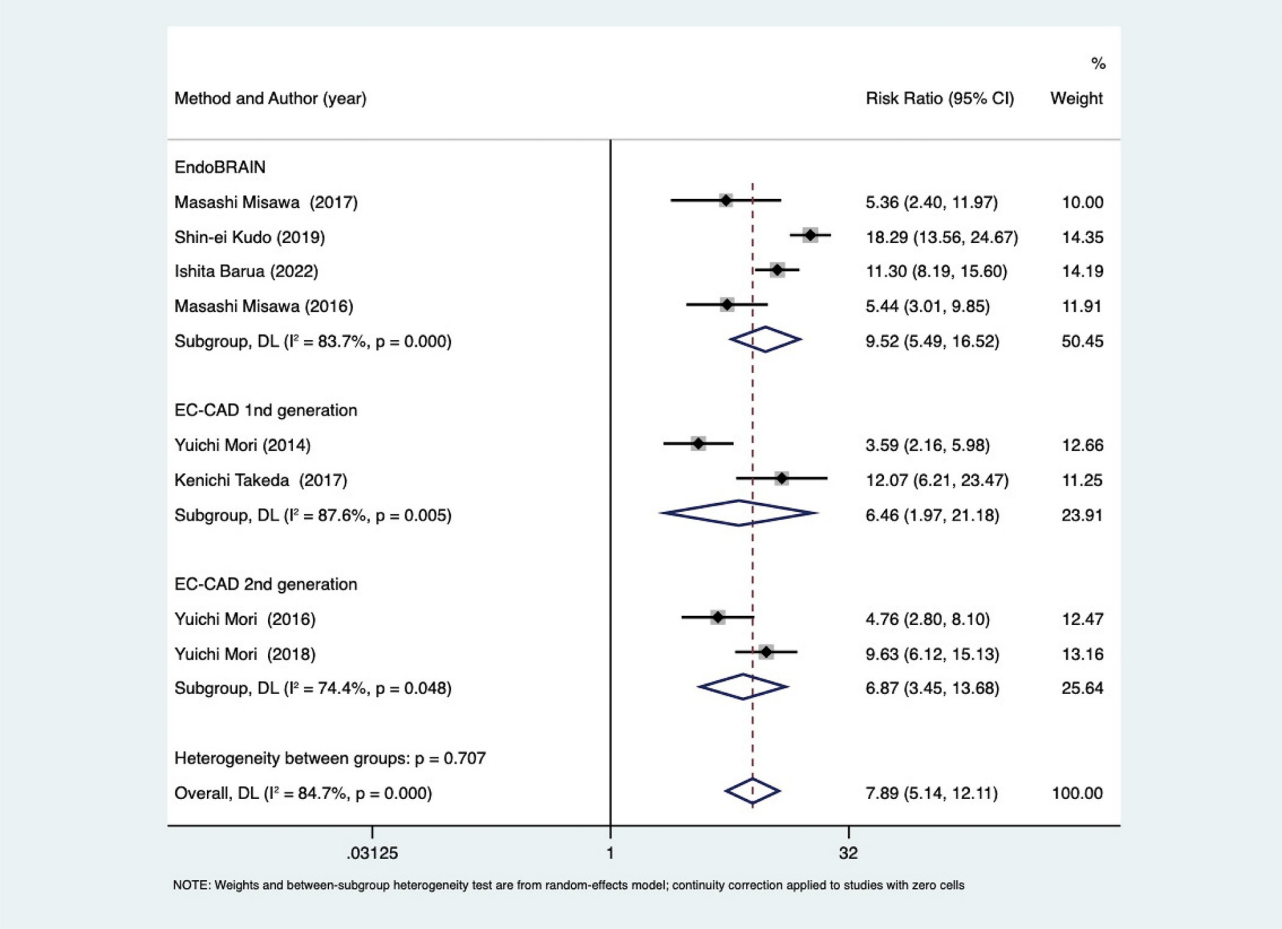
Illustrates the subgroup analysis of several artificial intelligence (AI) technologies.

The I^2^-test data for the pooled sensitivity and specificity was 87.98% (P < 0.05) and 95.60% (P < 0.05), 92.57% (P < 0.05), and 95.53% (P < 0.05), 91.48% (P < 0.05) and 93.82% (P < 0.05), respectively. The study employed subgroup and univariate meta-regression analyses to investigate potential factors contributing to heterogeneity. The covariates included in the meta-regression analysis consisted of study type (retrospective or prospective), study protocol (EndoBRAIN® or EC-CAD), sample size (≥200 or <200 lesions), magnification (520X or 380X), and NBI technology (no use or use). (Fig 7) demonstrates a potential association between NBI technology and variations in both sensitivity and specificity (Table 4). Subsequently, the EC-CAD 1st generation, EC-CAD 2nd generation, and EndoBrain were subjected to subgroup analysis. As mentioned above, the findings revealed significant heterogeneity amongst the groups, with a p-value of less than 0.05 (Fig 8).

**Table 4:** Presents the findings of the univariate meta-regression and subgroup analyses conducted to evaluate the accuracy of diagnosing colorectal lesions.

Parameter	category	No. of studies	Sensitivity	p1	Specificity	p2
study type	yes	5	0.91[0.88–0.94]	0.00	0.91[0.78–1.00]	0.62
	no	3	0.95[0.93–0.97]	.	0.98[0.95–1.00]	.
method	yes	4	0.93[0.90–0.96]	0.00	0.97[0.91–1.00]	0.09
	no	4	0.92[0.88–0.96]	.	0.93[0.81–1.00]	.
case	yes	5	0.90[0.87–0.94]	0.00	0.93[0.81–1.00]	0.95
	no	3	0.94[0.92–0.97]	.	0.98[0.93–1.00]	.
magnification	yes	5	0.90[0.87–0.94]	0.00	0.93[0.81–1.00]	0.95
	no	3	0.94[0.92–0.97]	.	0.98[0.93–1.00]	.
NBI	yes	6	0.93[0.91–0.96]	0.00	0.97[0.92–1.00]	0.03
	no	2	0.90[0.85–0.96]	.	0.87[0.57–1.00]	.

### Risk of bias assessment

**Fig 9:**
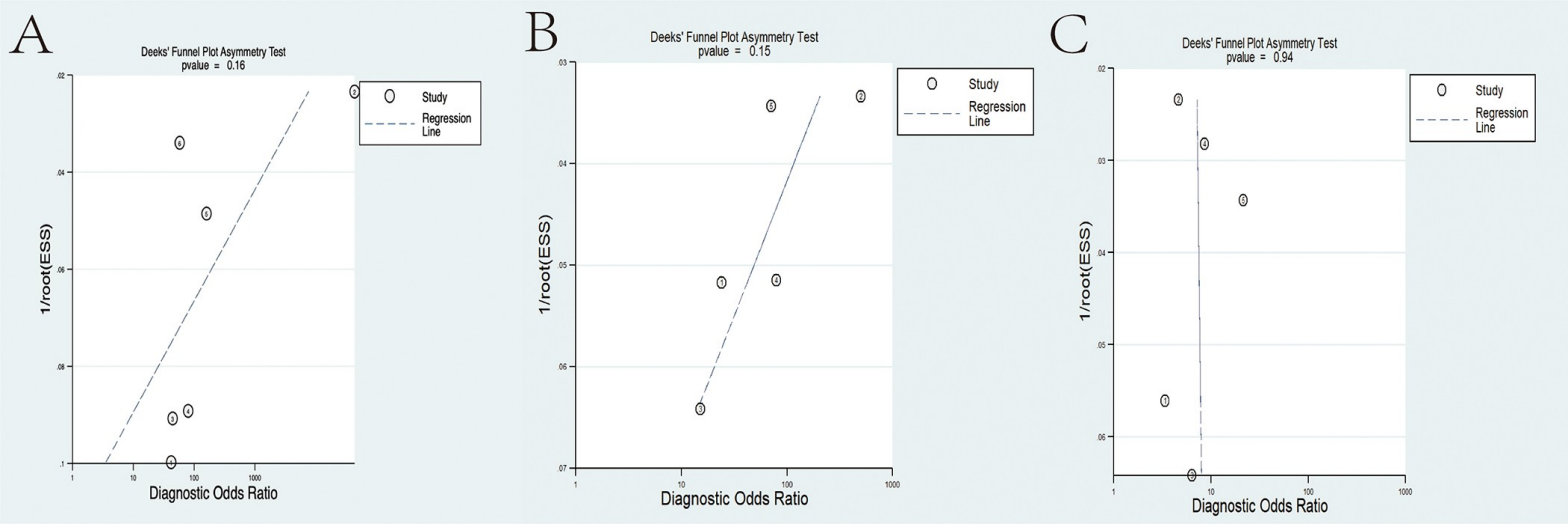
Deeks’ funnel plot of endocytoscopy using AI in colorectal lesions. A, AI group. B, expert group. C, trainee group.

In order to examine the phenomenon of publication bias, the researchers conducted a study using Deek’s funnel plot asymmetry method. The obtained results, specifically the P = 0.16, 0.15, and 0.94, indicated the absence of any noticeable asymmetry or publication bias in the funnel plot. These findings support the reliability and validity of the meta-analysis results (Fig 9).

## Discussion

Colorectal cancer is the 3^rd^ most frequent malignancy in both genders and the 2^nd^ most frequent cause of death by cancer globally [31]. EC is a recently established endoscopic modality comprising a contact light microscope attached to a conventional colonoscopy. In 2015, Yuichi Mori et al. revealed a novel EC-CAD with 89% accuracy for differentiating neoplastic alterations at 0.3sec/lesion [25]. The examination of this effective methodology for colorectal lesions has generated considerable attention. The research aimed to explore the efficacy of artificial intelligence in endocytoscopy-based cancer lesion detection. This study is the inaugural meta-analysis-incorporating systematic review of RC conducted via artificial intelligence, as far as our comprehension is concerned.

The meta-analysis data of 8 selected articles, including 2984 patients (4241 lesions), indicated that the pooled sensitivity and specificity for EC via AI was 0.93 (95%CI: 0.90, 0.95) and 0.94 (95% CI: 0.73, 0.99), the AUSROC curve of CAD was 0.95 (95% CI: 0.93, 0.97). The I^2^-test data for the pooled sensitivity = 87.98% (P < 0.05) and specificity = 95.53% (P < 0.05) was also observed. The diagnostic accuracy of the AI system surpassed that of trainee endoscopists and was comparable to that of experts. The sensitivity and specificity of the experts were determined to be 0.90 (95% CI: 0.85, 0.94) and 0.87 (95% CI: 0.78, 0.93), respectively, whereas the trainees exhibited a sensitivity of 0.74 (95% CI: 0.67, 0.79) and a specificity of 0.72 (95% CI: 0.62, 0.80). The AUSROC curve for specialists was determined to be 0.95 (95% CI: 0.93, 0.97), whereas for trainees, it was found to be 0.79 (95% CI: 0.75, 0.82). The accuracy of the diagnostic test ANOVA model showed a superior index of 4.00 (1.15,5.00), 2.00 (1.15,5.00), and 0.20 (0.20,0.20), respectively. There was significant heterogeneity among the three methods, for the I^2^-test pooled sensitivity and specificity were 89.59% (P < 0.05) and 95.60% (P < 0.05), 92.57% (P < 0.05) and 93.48% (P < 0.05), 91.48% (P < 0.05) and 93.82% (P < 0.05), respectively. The cause of potential heterogeneity was measured by subgroup and univariate meta-regression tests. The covariates included in the meta-regression analysis were study type (retrospective or prospective), study protocol (EndoBRAIN® or EC-CAD), sample size (≥200 or <200 lesions), magnification (520X or 380X), and NBI technology (no use or use). The association between NBI technology and the variability in sensitivity and specificity has been disclosed. There was no significant publication bias. This investigation revealed that AI could detect colorectal lesions with notable accuracy upon confident diagnosis. Furthermore, it had better diagnostic accuracy than endoscopists at the trainee level and was comparable to expert endoscopists. These results are consistent with the data of Cesare Hassan et al.. Evidence suggests that implementing AI to detect colorectal neoplasia can substantially increase the detection independent from main adenoma features [14].

Accurate diagnosis of colorectal lesions is paramount, as the complete removal of all adenomas significantly decreases the occurrence of malignancies and their corresponding mortality rates. Alessandro Repici et al. revealed that CAD could aid real-time colonoscopy and markedly increase adenoma identification per colonoscopy without elevated withdrawal time [32]. Michael B. Wallace et al. showed that AI produced approximately a 2-fold decrease in colorectal neoplasia miss rate. This suggests it reduces perceptual errors for diminutive and subtle lesions detected by standard colonoscopy [33]. According to Yasuharu Maeda et al. CAD system allow fully automated detection of persistent histologic inflammation linked with ulcerative colitis (UC) [34], and Takishima et al. revealed that the Goblet cells, when quantified by EC, suggested prolonged sustained UC patients’ clinical remission and that EC resembles histology more than endoscopy [35]. Julia Arribas et al. indicated an increased overall AI accuracy for diagnosing any UGI tract neoplastic lesion independent of the underlying state. This may substantially decrease precancerous lesions and early cancer miss rate in clinical practice [36]. Using an endocytoscope in conjunction with AI enables the real-time evaluation of microvascular and cellular histology of colorectal lesions. This technology significantly improves the diagnostic capabilities of endoscopists, leading to a notable increase in accuracy. This offers a significant advantage, particularly for endoscopists who lack expertise, as AI can equalize the situation by providing a standardized optical diagnostic method. Consequently, this can help minimize the impact of their poor knowledge. Increased time for acquiring endocytoscopic images is also a concern as the conventional procedure takes longer as the endoscopists need to position the endoscope on the lesion carefully, press the release button, and then check the computer diagnosis [37].

The National Institute for Health and Care Excellence (NICE), responsible for documenting clinical standards in England and Wales, has recently approved the optical identification of small colorectal polyps using narrow-spectrum endoscopy. This decision paves the way for the clinical adoption of this diagnostic approach [38]. Even though EC, separately or in combination with AI, can potentially provide significant diagnostic accuracy for distinguishing adenomas from hyperplastic polyps, it is cost- and resource-efficient. It should be implemented widely, and it is not because of a general challenge to widespread use [39]. Other obstacles may include limited accessibility and commercial availability of endocytoscopes. Currently, the EC-required 290-system is being utilized widely in the UK and Japan but is unavailable.

Another significant barrier is the regulatory approval for the global use of AI devices. The EndoBRAIN® tool, developed by Cybernet Systems Co., Ltd. in Tokyo, Japan, is a novel endocytoscopic artificial intelligence tool used for imaging. Its widespread approval is limited to Japan and some Asian nations [29]. Similarly, EndoBRAIN®-Plus identifies CRC was authorized in Japan (2020) [28]. The advancements made in Japan have motivated other countries to pursue the necessary regulatory authorizations. It is noteworthy to highlight that the utilization of AI technology produced an enhancement in physicians’ level of concern towards optical diagnostics. According to the findings of a survey, a considerable percentage of physicians, including 40%, reported experiencing discomfort when utilizing AI assistance. Additionally, this discomfort level experienced a substantial increase of 60% when physicians were equipped with AI tools that offered support [33]. Comprehensive research is needed to ascertain the perspectives of both medical practitioners and patients on AI [40].

This investigation was advantageous because: (1) it is the 1^st^ comprehensive research that investigates the role of EC with AI for diagnosing colorectal lesions;(2) it compares EC via AI with experts and trainees by ANOVA model, data was more reliable; (3) various databases were used for extensive searches, and numerous synonyms were linked. However, due to the meta-analysis restrictions, the limitations of this investigation are: (1) a relatively small number of clinical data on EC via AI for colorectal lesions was included; (2) selected articles were primarily published by Japanese scholars, presenting possible regional bias; (3) all the literature included was published in English; therefore, research data was limited, affecting its comprehensiveness; (4) possible high heterogeneity because of small study size.

## Conclusion

In summary, the findings of this meta-analysis suggest that the utilization of artificial intelligence in EC holds promise as a diagnostic tool for colorectal lesions. The utilization of this instrument has the potential to enhance the diagnostic process in routine EC procedures, as it has demonstrated superior diagnostic accuracy compared to trainee endoscopists and equivalent performance to professional endoscopists. However, this investigation presents limitations because of the reduced study size, regional bias, and different detection methods. Additional worldwide multicenter trials are necessary to validate the efficacy of this technology.

## Supporting information

S1 File (DOCX)

## Abbreviations

CRC: colorectal cancer
EC: endocytoscopy
AI: Artificial Intelligence
ECV-CAD: CAD system for endocytoscopic vascular pattern
CAD: computer-aided diagnosis
ASGE: American Society for Gastrointestinal Endoscopy
SSA/P: sessile serrated adenoma/polyp
NLR: negative likelihood ratio
PLR: positive likelihood ratio
SROC: summary receiver operator characteristic
AUC: Area Under Curve
ESGE: European Society of Gastrointestinal Endoscopy
