# Supplementary material for: Diagnostic accuracy of endocytoscopy via artificial intelligence in colorectal lesions: A systematic review and meta‑analysis
Source: PLoS One. 2023 Dec 19;18(12):e0294930. doi: 10.1371/journal.pone.0294930 (PMC10729963; doi:10.1371/journal.pone.0294930)
Supplement: S1 File — (DOCX) [file pone.0294930.s001.docx]

SUPPLEMENTARY MATERIALS OF

**Diagnostic accuracy of** **endocytoscopy via Artificial Intelligence in** **colorectal lesions: a systematic review and meta‑analysis**

1. The PRISMA 2020 statement.
2. Search strategy.
3. Results of individual studies.
4. Figure combining the published meta-analyses.
5. Accuracy of diagnostic test Results of mesh meta-analysis ANOVA model.
6. Registration information in PROSPERO.

| **Section** **and** **Topic** | **Item** **#** | **Checklist** **item** | **Location** **where** **item** **is** **reported** |
| --- | --- | --- | --- |
| **TITLE** | | |  |
| Title | 1 | Identify the report as a systematic review. | Page 1 |
| **ABSTRACT** | | |  |
| Abstract | 2 | See the PRISMA 2020 for Abstracts checklist. | Page 1 |
| **INTRODUCTION** | | |  |
| Rationale | 3 | Describe the rationale for the review in the context of existing knowledge. | Page 2-3 |
| Objectives | 4 | Provide an explicit statement of the objective(s) or question(s) the review addresses. | Page 2-3 |
| **METHODS** | | |  |
| Eligibility criteria | 5 | Specify the inclusion and exclusion criteria for the review and how studies were grouped for the syntheses. | Page 3 |
| Information sources | 6 | Specify all databases, registers, websites, organisations, reference lists and other sources searched or consulted to identify studies. Specify the date when each source was last searched or consulted. | Page 3 |
| Search strategy | 7 | Present the full search strategies for all databases, registers and websites, including any filters and limits used. | Page 3 |
| Selection process | 8 | Specify the methods used to decide whether a study met the inclusion criteria of the review, including how many reviewers screened each record and each report retrieved, whether they worked independently, and if applicable, details of automation tools used in the process. | Page 3 |
| Data collection process | 9 | Specify the methods used to collect data from reports, including how many reviewers collected data from each report, whether they worked independently, any processes for obtaining or confirming data from study investigators, and if applicable, details of automation tools used in the process. | Page 3 |
| Data items | 10a | List and define all outcomes for which data were sought. Specify whether all results that were compatible with each outcome domain in each study were sought (e.g. for all measures, time points, analyses), and if not, the methods used to decide which results to collect. | Page 3 |
|  | 10b | List and define all other variables for which data were sought (e.g. participant and intervention characteristics, funding sources). Describe any assumptions made about any missing or unclear information. | Page 3 |
| Study risk of bias  assessment | 11 | Specify the methods used to assess risk of bias in the included studies, including details of the tool(s) used, how many reviewers assessed each study and whether they worked independently, and if applicable, details of automation tools used in the process. | Page 4 |
| Effect measures | 12 | Specify for each outcome the effect measure(s) (e.g. risk ratio, mean difference) used in the synthesis or presentation of results. | Page 4 |
| Synthesis methods | 13a | Describe the processes used to decide which studies were eligible for each synthesis (e.g. tabulating the study intervention characteristics and comparing against the planned groups for each synthesis (item #5)). | Page 4 |
|  | 13b | Describe any methods required to prepare the data for presentation or synthesis, such as handling of missing summary statistics, or data conversions. | Page 4 |
|  | 13c | Describe any methods used to tabulate or visually display results of individual studies and syntheses. | Page 4 |
|  | 13d | Describe any methods used to synthesize results and provide a rationale for the choice(s). If meta-analysis was performed, describe the model(s), method(s) to identify the presence and extent of statistical heterogeneity, and software package(s) used . | Page 4 |
|  | 13e | Describe any methods used to explore possible causes of heterogeneity among study results (e.g. subgroup analysis, meta-regression). | Page 4 |
|  | 13f | Describe any sensitivity analyses conducted to assess robustness of the synthesized results. | Page 4 |
| Reporting bias  assessment | 14 | Describe any methods used to assess risk of bias due to missing results in a synthesis (arising from reporting biases). | Page 3-4 |

| **Section** **and** **Topic** | **Item** **#** | **Checklist** **item** | **Location** **where** **item** **is** **reported** |
| --- | --- | --- | --- |
| Certainty assessment | 15 | Describe any methods used to assess certainty (or confidence) in the body of evidence for an outcome. | Page 3-4 |
| **RESULTS** | | |  |
| Study selection | 16a | Describe the results of the search and selection process, from the number of records identified in the search to the number of studies included in the review, ideally using a flow diagram. | Page 4-5 |
|  | 16b | Cite studies that might appear to meet the inclusion criteria, but which were excluded, and explain why they were excluded. | Page 4-5 |
| Study characteristics | 17 | Cite each included study and present its characteristics. | Page 5 |
| Risk of bias in studies | 18 | Present assessments of risk of bias for each included study. | Page 5 |
| Results of individual studies | 19 | For all outcomes, present, for each study: (a) summary statistics for each group (where appropriate) and (b) an effect estimate and its precision (e.g. confidence/credible interval), ideally using structured tables or plots. | Page 5 |
| Results of syntheses | 20a | For each synthesis, briefly summarise the characteristics and risk of bias among contributing studies. | Page 5-6 |
|  | 20b | Present results of all statistical syntheses conducted. If meta-analysis was done, present for each the summary estimate and its precision (e.g . confidence/credible interval) and measures of statistical heterogeneity. If comparing groups, describe the direction of the effect. | Page 5-6 |
|  | 20c | Present results of all investigations of possible causes of heterogeneity among study results. | Page 5-6 |
|  | 20d | Present results of all sensitivity analyses conducted to assess the robustness of the synthesized results. | Page 5-6 |
| Reporting biases | 21 | Present assessments of risk of bias due to missing results (arising from reporting biases) for each synthesis assessed. | Page 5-6 |
| Certainty of evidence | 22 | Present assessments of certainty (or confidence) in the body of evidence for each outcome assessed. | Page 5-6 |
| **DISCUSSION** | | |  |
| Discussion | 23a | Provide a general interpretation of the results in the context of other evidence. | Page 6-6 |
|  | 23b | Discuss any limitations of the evidence included in the review. | Page 8-9 |
|  | 23c | Discuss any limitations of the review processes used. | Page 8-9 |
|  | 23d | Discuss implications of the results for practice, policy, and future research. | Page 8-9 |
| **OTHER** **INFORMATION** | | |  |
| Registration and  protocol | 24a | Provide registration information for the review, including register name and registration number, or state that the review was not registered. | Page 3 |
|  | 24b | Indicate where the review protocol can be accessed, or state that a protocol was not prepared. | Page 3 |
|  | 24c | Describe and explain any amendments to information provided at registration or in the protocol. | Page 3 |
| Support | 25 | Describe sources of financial or non-financial support for the review, and the role of the funders or sponsors in the review. | Page 10 |
| Competing interests | 26 | Declare any competing interests of review authors. | Page 10 |
| Availability of data,  code and other  materials | 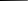27 | 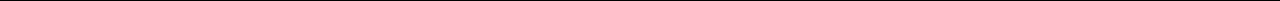Report which of the following are publicly available and where they can be found: template data collection forms; data extracted from included studies; data used for all analyses; analytic code; any other materials used in the review. | Page 10 |

*From:* Page MJ, McKenzie JE, Bossuyt PM, et al. The PRISMA 2020 statement: an updated guideline for reporting systematic reviews. *BMJ* 2021;372:n71. doi:10. 1136/bmj.n71

**Search strategy**

PubMed.

| 1. "Artificial Intelligence"[MeSH Terms] OR "diagnosis, computer assisted"[MeSH Terms] OR "Artificial Intelligence"[Title/Abstract] OR "AI"[Title/Abstract] OR "deep learning"[Title/Abstract] OR "machine learning"[Title/Abstract] OR "computer aided"[Title/Abstract] OR "computer-aided"[Title/Abstract] OR "neural network"[Title/Abstract] OR "CNN"[Title/Abstract] OR "automat*"[Title/Abstract] OR "CAD"[Title/Abstract] |
| --- |
| 1. "endocytoscopy"[Title/Abstract] OR "endocytoscopes"[Title/Abstract] OR "EC*"[Title/Abstract] |
| 1. ("Colorectal Neoplasms"[MeSH Terms] OR "neoplas*"[Title/Abstract] OR "tumor*"[Title/Abstract] OR "cancer*"[Title/Abstract] OR "carcinoma*"[Title/Abstract] OR "Intestinal Polyps"[MeSH Terms] OR "lesion*"[Title/Abstract] OR "polyp*"[Title/Abstract]) AND "colo*"[Title/Abstract] |
| 1. #1 and #2 and #3 |
| There were 57 records identified through PubMed database searching.  EMBASE. |
| 1. (Artificial Intelligence or AI or deep learning or machine learning or computer aided or computer-aided or neural network or CNN or automat* or CAD).ab,ti,kw. |
| 1. (endocytoscopy or endocytoscopes or EC*).ab,ti,kw. |
| 1. ((Colorectal Neoplasms or neoplas* or tumor* or cancer* or carcinoma* or Intestinal Polyps or lesion* or polyp*) and colo*).ab,ti,kw. |
| 1. #1 and #2 and #3 |
| There were 64 records identified through EMBASE database searching  Web of Science |
| 1. TS=(Artificial Intelligence or AI or deep learning or machine learning or computer aided or computer-aided or neural network or CNN or automat* or CAD) |
| 1. TS=(endocytoscopy or endocytoscopes or EC*) |
| 1. TS=((Colorectal Neoplasms or neoplas* or tumor* or cancer* or carcinoma* or Intestinal Polyps or lesion* or polyp*) and colo*) |
| 1. #1 and #2 and #3 |
| There were 63 records identified through Web of Science database searching. |
| Cochrane Library |

#1 (artificial intelligence):ab,ti,kw OR (AI):ab,ti,kw OR (deep learning):ab,ti,kw OR (machine learning):ab,ti,kw OR (computer aided):ab,ti,kw OR (computer-aided):ab,ti,kw OR (neural network):ab,ti,kw OR (CNN):ab,ti,kw OR (automat*):ab,ti,kw OR (CAD):ab,ti,kw : 31383

#2 (endocytoscopy):ab,ti,kw OR (endocytoscopes):ab,ti,kw OR (EC*):ab,ti,kw

#3 ((neoplas*):ab,ti,kw OR (tumor*):ab,ti,kw OR (cancer*):ab,ti,kw OR (carcinoma*):ab,ti,kw OR (lesion*):ab,ti,kw OR (polyp*):ab,ti,kw) AND (colo*):ab,ti,kw

#4 #1 AND #2 AND #3

There were 32 records identified through Cochrane Library database searching

CNKI

(主题:("人工智能" or "计算机辅助检测" or "深度学习" or "卷积神经网络" or "机器学习" or "神经网络") and 主题:("结直肠息肉" or "结直肠腺瘤" or "结直肠病变" or "锯齿状病变") and 主题:("细胞内镜") )

| Author | Year | Country | Study type | No. of lesions | Age | Sex male% | Size (mm) | Location (n) | Shape | Histopathology |  |
| --- | --- | --- | --- | --- | --- | --- | --- | --- | --- | --- | --- |
| Masashi Misawa | 2017 | Japan | Retrospective | 124 | 65.2 ± 10.6 | 36/58 | 8.7 ± 8.8 | Right 27；Left 25；Rectum 11 | Protruded 20；Flat 37；Depressed 4；Type 2 2 | Non-neoplastic Hyperplastic polyp 15; Low-grade adenoma 39  NeoplasticHigh-grade adenoma 3 ; Invasive cancer 7 |  |
| Shin-ei Kudo | 2019 | Japan | Prospective | 2000 | 66.3 (9.8) | 63/89 | 4（3-5） | Right colon 38 (38.0); Left colon 30 (30.0); Rectum 32 (32.0) | Polypoid (Is, Ip) 40 (40.0); Slightly elevated (IIa) 60 (60.0) | Non-neoplastic, n (%) Hyperplastic polyp 34 (34.0); Inflammatory polyp 1 (1.0)  Neoplastic, n (%)Tubular adenoma 63 (63.0); Tubulo-villous adenoma 2 (2.0) |  |
| Yuichi Mori | 2014 | Japan | Retrospective | 176 | 64.2±12.1 | 107/152 | 6.3 (2.4) | Right colon 99（48);Left colon 73 (36);Rectum 33 (16) | Polypoid (Is, Ip) 97 (55.2); Slightly elevated (IIa) 71 (40.3); Slightly depressed (IIc, IIaþIIc) 8 (4.5) | Non-neoplastic, Hyperplastic polyp 30 (17.0); Inflammatory polyp 5 (2.8); Juvenile polyp 4 (2.3)  Neoplastic, Low-grade adenoma 104 (59.1); High-grade adenoma 26 (14.8); Invasive cancer 7 (4.0) |  |
| Yuichi Mori | 2016 | Japan | Prospective | 139 | 65(10) | 84/134 | 5(2) | Right colon 77（43.7);Left colon 66 (37.5);Rectum 33 (18.8) | Polypoid (Is, Ip) 86 (42); Slightly elevated (IIa) 112(55); Slightly depressed (IIc, IIaþIIc) 7 (3) | Non-neoplastic, Hyperplastic polyp 30 (17.0); Inflammatory polyp 5 (2.8); Juvenile polyp 4 (2.3)  Neoplastic, Low-grade adenoma 104 (59.1); High-grade adenoma 26 (14.8); Invasive cancer 7 (4.0) |  |
| Yuichi Mori | 2018 | Japan | Prospective | 450 | 67 (58–73) | 235/325 | 3（3-4） | Cecum 28 (6.0); Ascending colon 81 (17.4); Transverse colon 78 (17.0); Descending colon 29 (6.2); Sigmoid colon 137 (29.4); Rectum 113 (24.2) | Polypoid (Is and Ip) 105 (22.5); Slightly elevated (IIa) 360 (77.3); Slightly depressed (IIc) 1 (0.2) | Neoplastic 282 (60.5); Nonneoplastic 176 (37.8); Nonanalyzable† 8 (1.7) |  |
| Kenichi Takeda | 2017 | Japan | Retrospective | 375 | Adenoma 65.3 ± 11.7  Invasive cancer 64.2 ± 11.2 | 45/76 | Adenoma 11.0 ± 9.5  Invasive cancer 30.8 ± 14.3 | Cecum 7; Ascending colon 9; Transverse colon 19; Descending colon 6; Sigmoid colon 24; Rectum 11 | Polypoid (Is, Isp, Ip) 36; Slightly elevated (IIa); 35 Slightly depressed (IIc, IIa +IIc, Is +IIc)5 | Low grade adenoma 48; High grade adenoma 6; Invasive cancer 22 |  |
| Masashi Misawa | 2016 | Japan | Retrospective | 85 | 63.8±12.0 | 20/33 | 8.6 ± 10.3 | Right colon 15; Left colon 10; Rectum 11 | Protruded 9; Flat elevated 26; Depressed 1 | Nonneoplasms, Hyperplastic polyp 17  Neoplasms, Low-grade adenoma 14; High-grade adenoma 3; Invasive carcinoma 2 |  |
| Ishita Barua | 2022 | Japan | Prospective | 892 | 67 (60–74) | 327/518 | Neoplastic Polyps 4(3-5)  Nonneoplastic Polyps 3（2-3) | Neoplastic, Sigmoid colon 274 (76.3);Rectum 85 (23.7)  Nonneoplastic, Sigmoid colon 260 (48.8);Rectum 273 (51.2) | Neoplastic, Polypoid (Is, Ip) 175 (48.7);Nonpolypoid (type IIa) 184 (51.3)  Nonneoplastic, Polypoid (Is, Ip) 109 (20.5);Nonpolypoid (type IIa) 424 (79.5) | Non-neoplastic, Hyperplastic polyp 485 (91.0); Inflammatory polyp 8 (1.5); Other 40 (7.5)  Neoplastic, Low-grade adenoma 335 (93.3); High-grade adenoma 5 (1.4); sessile serrated 19 (5.3) |  |

Results of individual studies.

The diagnostic accuracy data of endocytoscopy using Artificial Intelligence in colorectal lesions.

| Author | Magnification | Scope | Method | CAD | | | | Experts | | | | Trainees | | |  |
| --- | --- | --- | --- | --- | --- | --- | --- | --- | --- | --- | --- | --- | --- | --- | --- |
|  |  |  |  | TP | FP | FN | TN | TP | FP | FN | TN | TP | FP | FN | TN |
| Masashi Misawa | 380X | NBI | EndoBRAIN® | 84 | 10 | 5 | 25 | 318 | 25 | 53 | 100 | 158 | 37 | 99 | 78 |
| Shin-ei Kudo | 520X | NBI | EndoBRAIN® | 1260 | 0 | 40 | 700 | 603 | 20 | 20 | 330 | 920 | 240 | 380 | 460 |
| Yuichi Mori | 380X | WLI | EC-CAD 1st | 126 | 8 | 11 | 31 | 242 | 26 | 32 | 52 | 228 | 34 | 46 | 44 |
| Yuichi Mori | 380X | WLI | EC-CAD 2nd | 80 | 4 | 11 | 44 | 248 | 16 | 25 | 128 | 646 | 106 | 264 | 374 |
| Yuichi Mori | 520X | NBI | EC-CAD 2nd | 262 | 15 | 17 | 156 | 476 | 26 | 82 | 316 | 439 | 50 | 119 | 292 |
| Kenichi Takeda | 380X | NBI | EC-CAD 1st | 68 | 1 | 8 | 90 |  |  |  |  |  |  |  |  |
| Masashi Misawa | 380X | NBI | EndoBRAIN® | 49 | 1 | 9 | 41 |  |  |  |  |  |  |  |  |
| Ishita Barua | 520X | NBI | EndoBRAIN® | 335 | 74 | 35 | 448 |  |  |  |  |  |  |  |  |

Figure

**
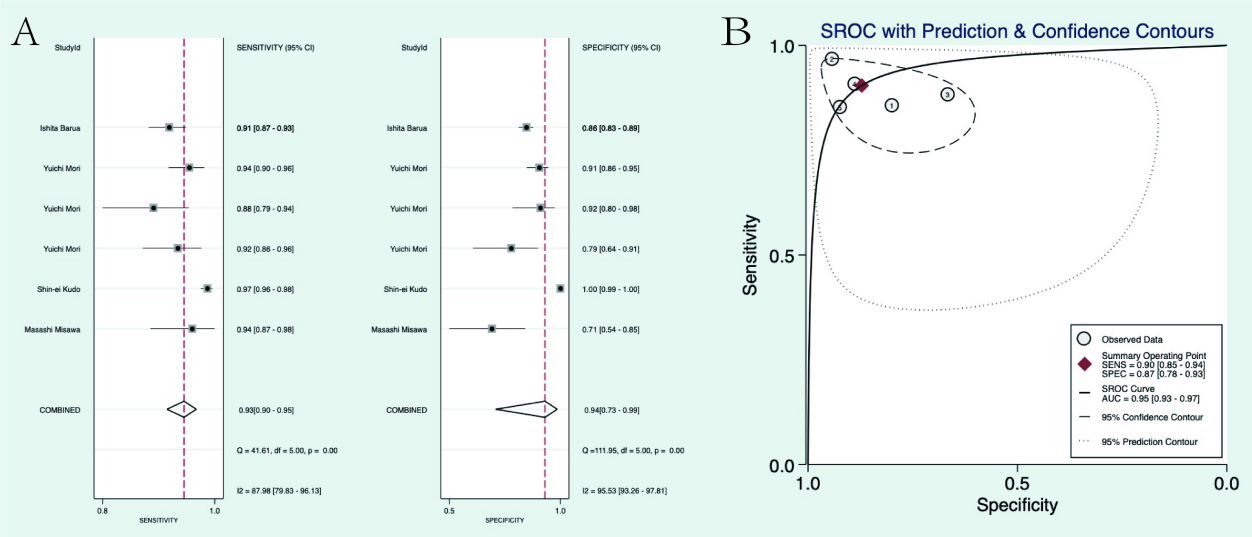
**


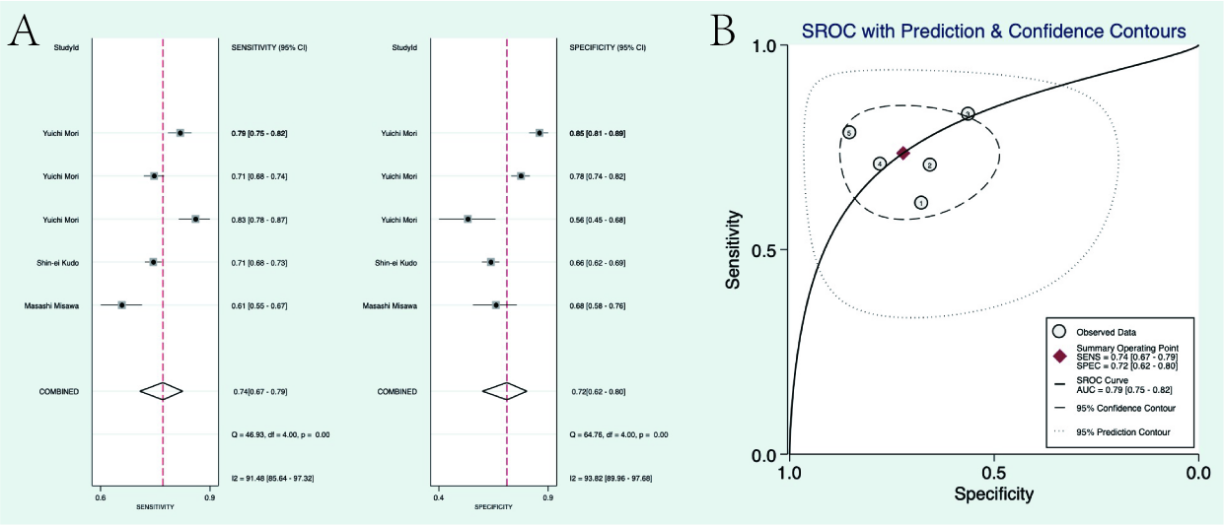

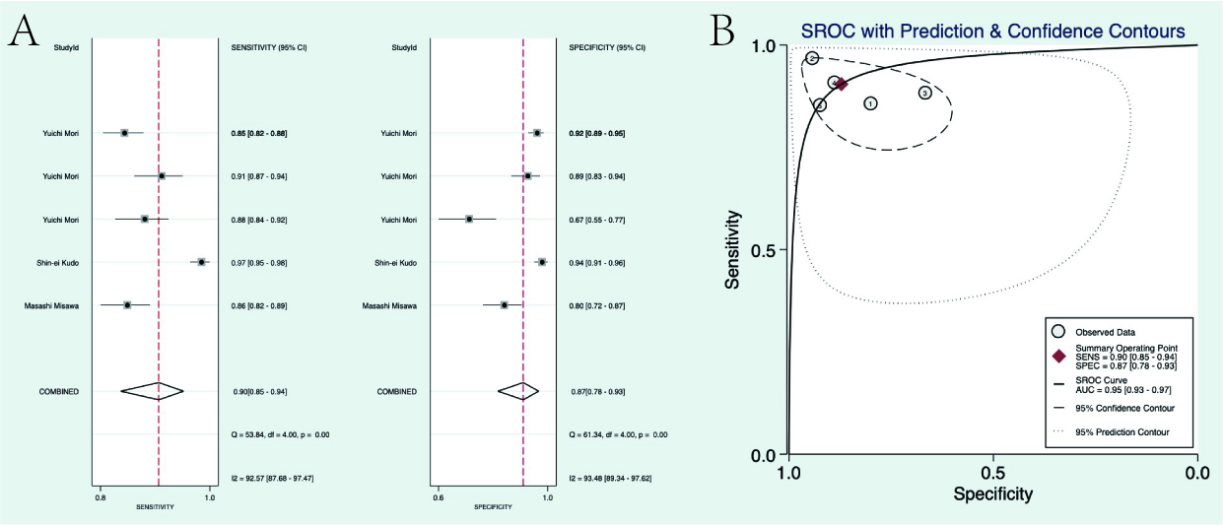


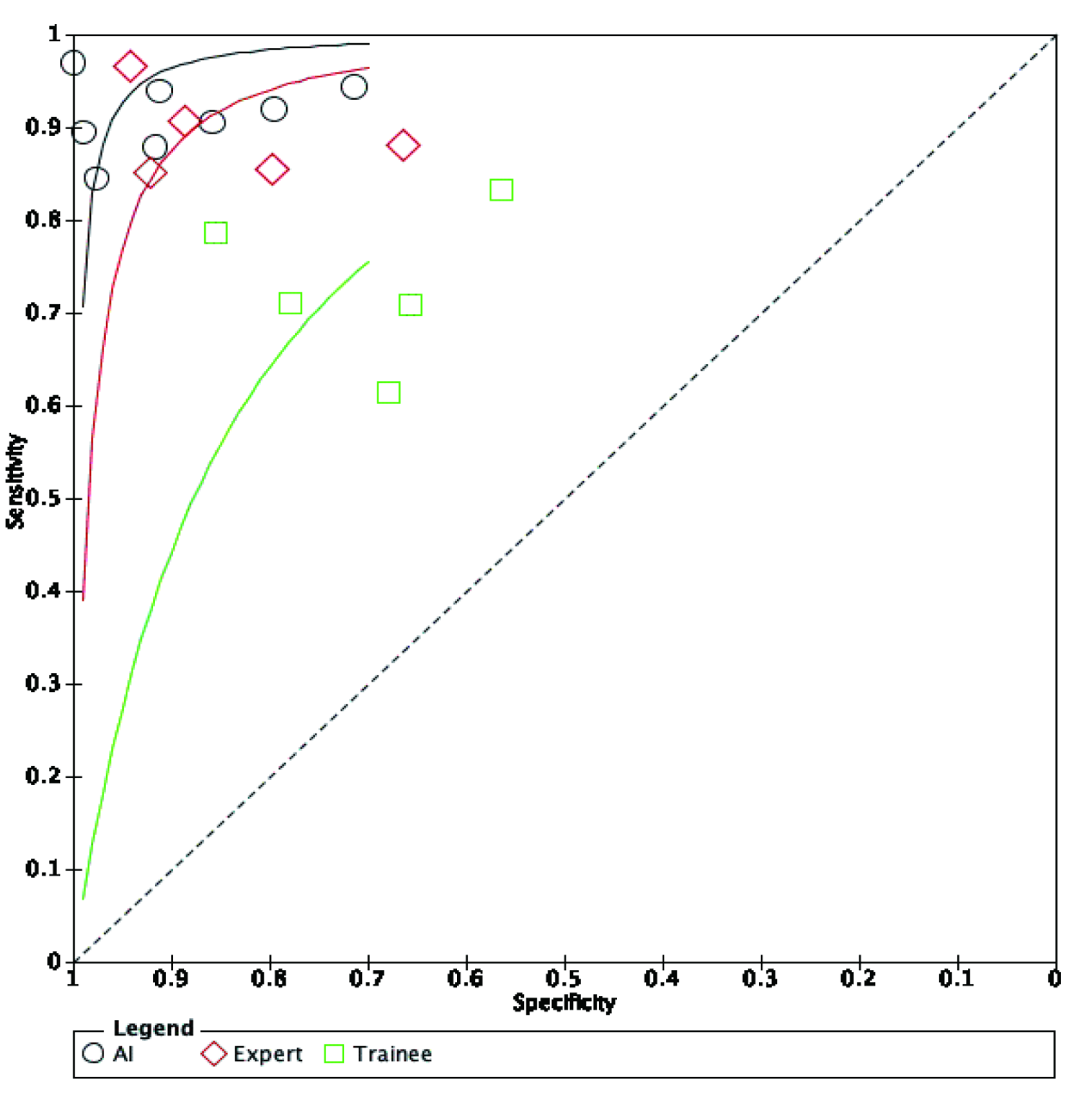


ANOVA model


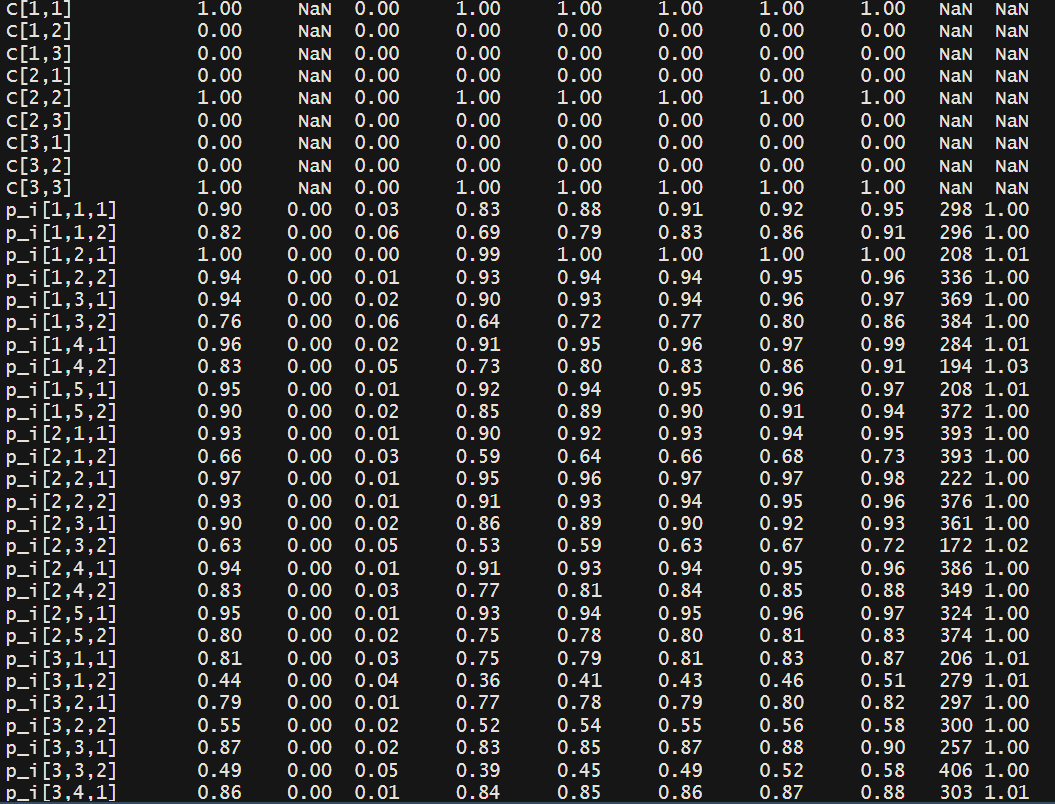

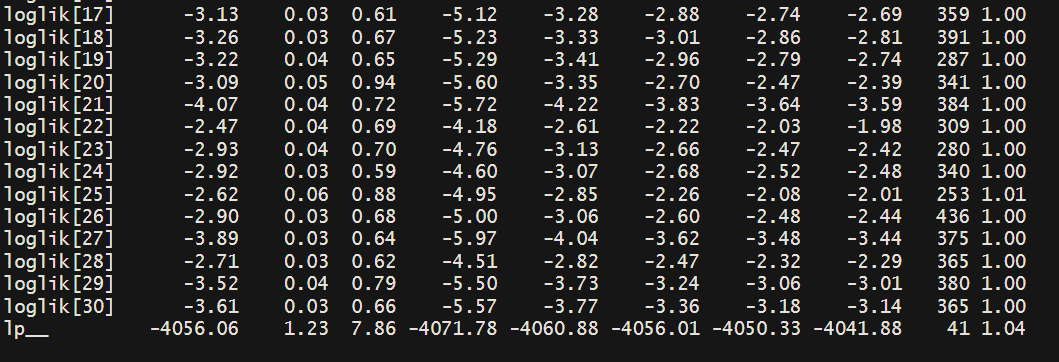

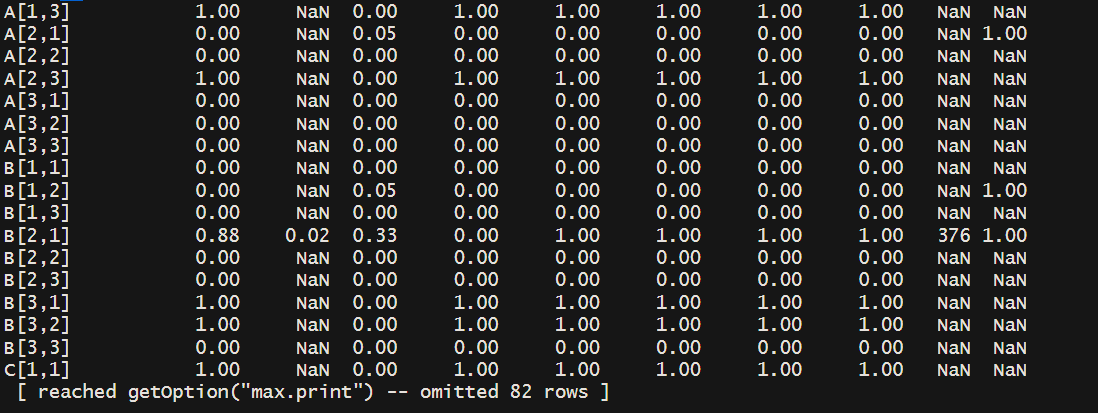


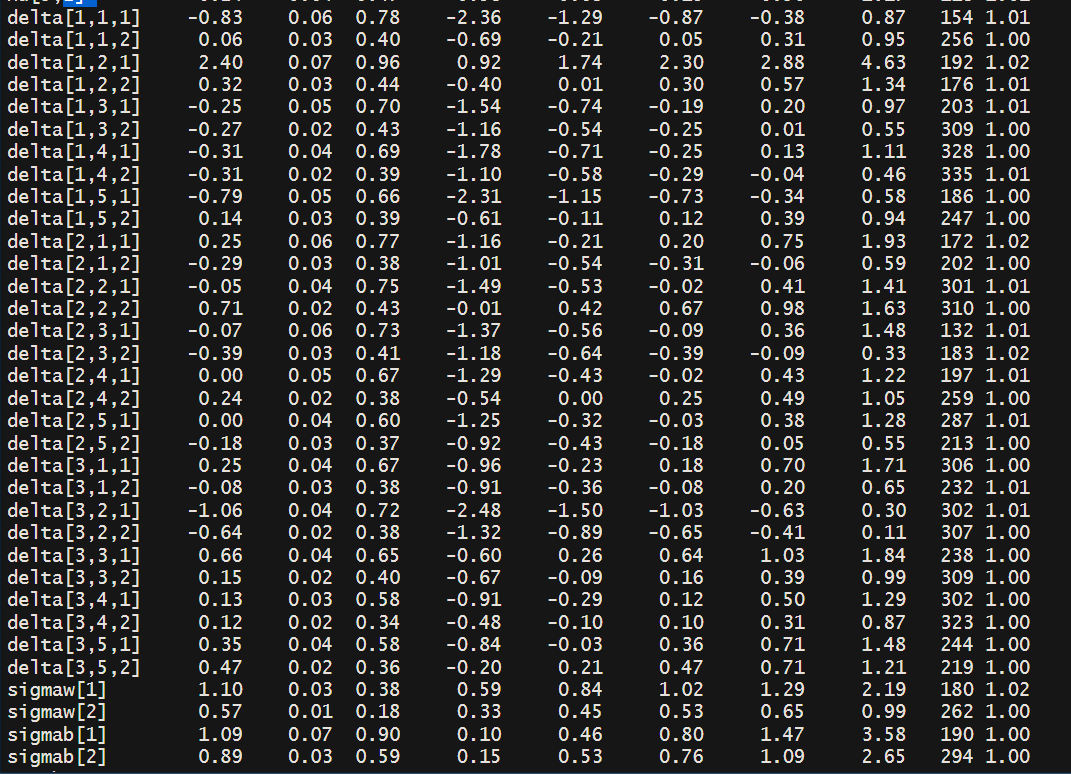

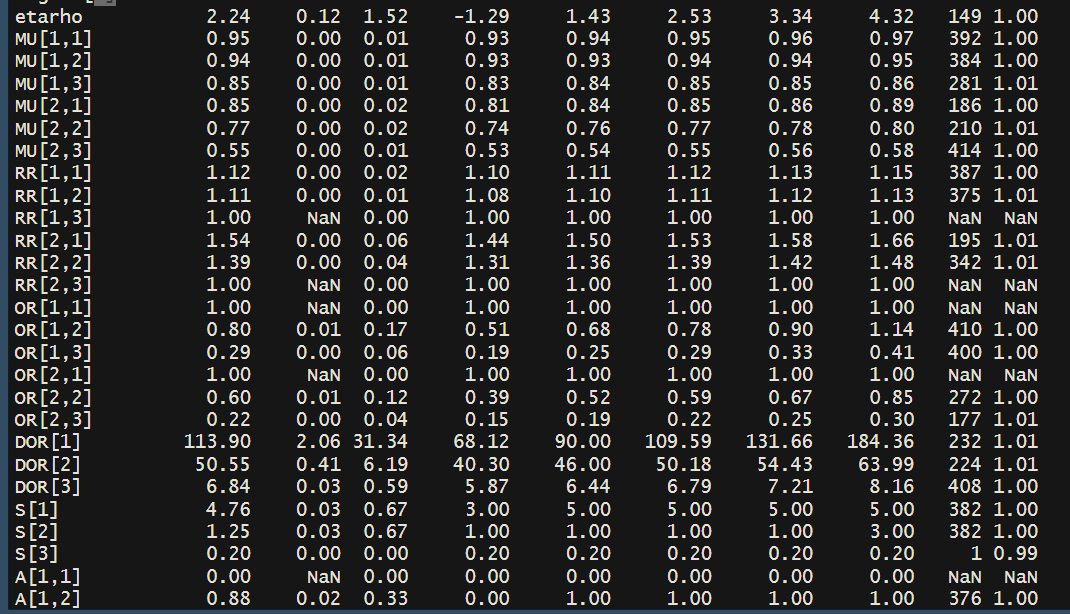

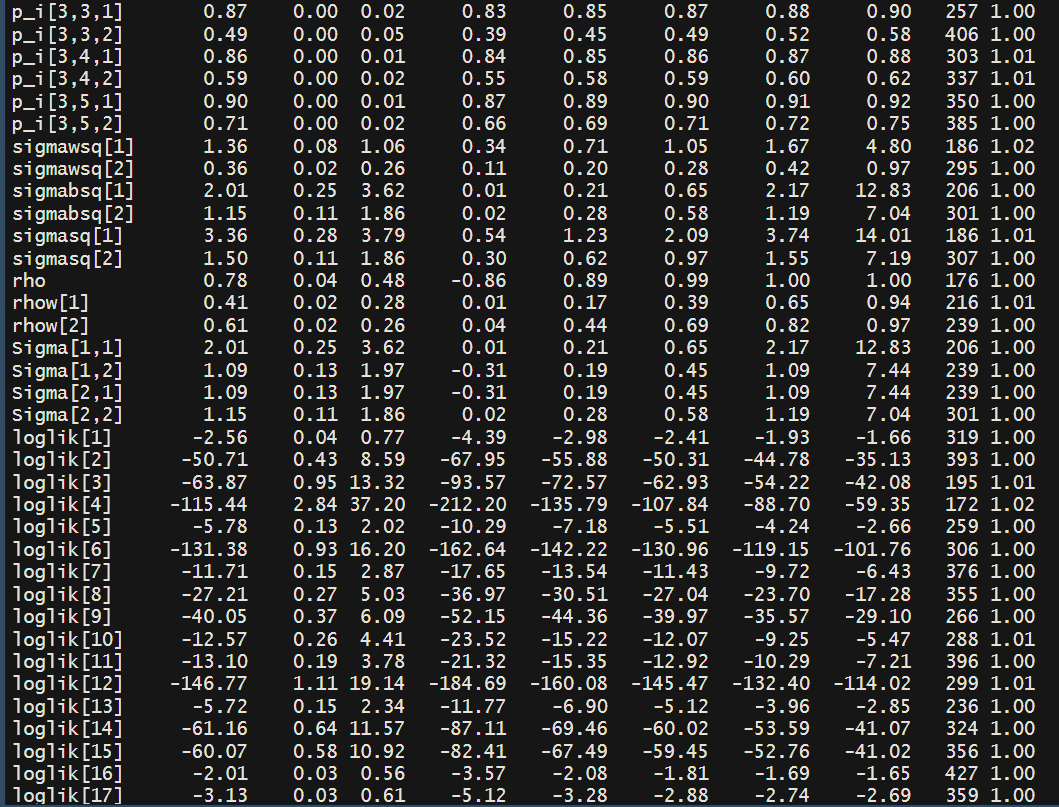

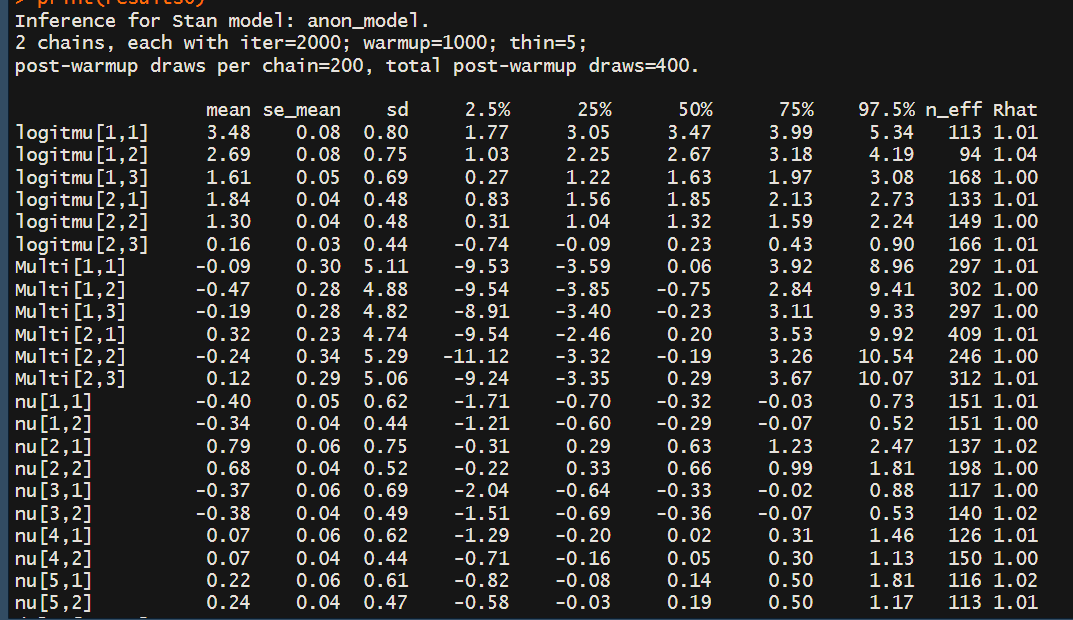


PROSPERO

https://www.crd.york.ac.uk/prospero/display_record.php?ID=CRD42023388421
